# Graphene Oxide-Based Membranes Intercalated with an Aromatic Crosslinker for Low-Pressure Nanofiltration

**DOI:** 10.3390/membranes12100966

**Published:** 2022-10-02

**Authors:** Hyuntak Kwon, Yongju Park, Euntae Yang, Tae-Hyun Bae

**Affiliations:** 1 Department of Chemical and Biomolecular Engineering, Korea Advanced Institute of Science and Technology (KAIST), 291 Daehak-ro, Yuseong-gu, Daejeon 34141, Korea; 2 Department of Marine Environmental Engineering, College of Marine Science, Gyeongsang National University, Tongyeong 53064, Korea

**Keywords:** graphene oxide, interlayer spacing, nanofiltration, desalination, molecular separation

## Abstract

Graphene oxide (GO), a carbonaceous 2D nanomaterial, has received significant interest as a next-generation membrane building block. To fabricate high-performance membranes, an effective strategy involves stacking GO nanosheets in laminated structures, thereby creating unique nanochannel galleries. One outstanding merit of laminar GO membranes is that their permselectivity is readily tunable by tailoring the size of the nanochannels. Here, a high-performance GO-based nanofiltration membrane was developed by intercalating an aromatic crosslinker, α,α/-dichloro-p-xylene (DCX), between the layers in laminated GO nanosheets. Owing to the formation of strong covalent bonds between the crosslinker and the GO, the resulting GO laminate membrane exhibited outstanding structural stability. Furthermore, due to the precisely controlled and enlarged interlayer spacing distance of the developed DCX-intercalated GO membrane, it achieved an over two-fold enhancement in water permeability (11 ± 2 LMH bar^−1^) without sacrificing the rejection performance for divalent ions, contrary to the case with a pristine GO membrane.

## 1. Introduction

Over the last few decades, the acceleration of global water scarcity by climate change has necessitated the development of technologies that can produce clean water from alternative water resources, such as seawater and wastewater. Among those technologies, membrane-based separations have played a pivotal role in the conversion of alternative water resources into clean water [1,2,3,4,5]. In particular, low-pressure nanofiltration (NF) is a highly useful and applicable process that offers low energy requirements and excellent removal efficiencies for most contaminants, including divalent ions [6,7]. Currently, polymeric membranes are commonly used for NF because of their excellent economic viability and processability [8,9,10]. However, polymer-based membranes generally suffer from restricted separation performance because of their inherent limitations, including their chaotic water transport channels, low mechanical and chemical stabilities under certain conditions, and empirical fabrication approach without molecular-level design [11,12,13]. Therefore, to overcome these innate shortcomings and, consequently, fabricate high-performance NF membranes, new membrane materials are required as replacements for polymers.

In this regard, graphene oxide (GO) nanosheets, 2D nanomaterials consisting of sp^2^ carbon atoms in a hexagonal lattice structure with oxygen functional groups, have been extensively investigated as feasible materials for designing innovative NF membranes [14,15]. GO nanosheets have several merits as NF membrane materials, such as their high atomic thickness, strong hydrophilicity, good processability, high aspect ratio, good chemical tunability, and amphipathic property [16,17,18]. Considering these excellent properties, the vertical stacking of GO nanosheets in a laminated structure is a good strategy for designing GO-based membranes. In stacked GO laminates, unique nanochannel networks, consisting of hydrophilic regions acting as pillars for securing space and hydrophobic regions acting as routes for mass transport, are created [19,20]. These unique nanochannel networks can offer the unprecedentedly ultrafast transport of water molecules as well as the precise sieving of molecules based on size exclusion [11,21,22,23]. Furthermore, the size of the nanochannels can be easily tuned by intercalating specific sizes of crosslinkers, which can promote the fabrication of a broad spectrum of membranes with different nanochannel sizes. For example, by intercalating nanofibers in between layered GO nanosheets, GO membranes with nanochannel sizes larger than 2 nm can be produced [24,25,26]. When monomers or polymers are crosslinked between stacked GO nanosheets, GO nanochannels with sizes of 1–2 nm are fabricated. Even smaller GO nanochannels are obtained through the chemical reduction of GO laminates or the insertion of alkali cations, such as potassium ions, into the GO laminates [13,27]. Compared to conventional polymeric membranes, the GO membranes whose nanochannels are tailored to specific sizes have a narrow pore-size distribution, which is advantageous for improving the molecular weight cutoff.

Here, we employed a nonpolar organic compound, α,α/-dichloro-p-xylene (DCX), as a crosslinker to fabricate robust GO-based loose NF membranes with high molecular cutoff values for the efficient treatment of organic dye wastewater [28,29]. To the best of our knowledge, DCX and similar alkyl halide-bearing reagents had not yet been employed in the fabrication of GO laminate membranes. It was expected that DCX would react with the oxygen functional groups of GO and, consequently, yield highly interlocked stacked GO layers. Moreover, the relatively lower cost of DCX among similar molecules with various halide groups will enable the fabrication of cost-efficient GO-based NF membranes [30]. Through the careful optimization of the fabrication conditions, we successfully realized DCX-crosslinked GO (GO/DCX) NF membranes that outperformed the existing membranes. The developed GO/DCX membranes demonstrated high rejection efficiencies toward organic dyes and divalent ions (up to ~100%) as well as an excellent water permeability of up to ~11 LMH bar^−1^. In addition, during the long-term operation of the developed GO/DCX NF membranes (120 h), a highly stable organic dye rejection efficiency of >98% was achieved consistently without any performance deterioration.

## 2. Materials and Methods

### 2.1. Materials

For the synthesis of GO, graphite powders (carbon basis: 99.0%, particle size: ~325 mesh), potassium persulfate (99.0%), and phosphorus pentoxide (99.0%) were purchased from Sigma-Aldrich (St. Louis, MO, US) and potassium permanganate (99.3%) was purchased from Junsei Chemical (Tokyo, Japan). Sulfuric acid (95.0%), hydrogen peroxide (30.0%), and hydrochloric acid (35.0%) were purchased from Daejung Chemical (Si-heung, Korea). DCX (98.0%) and methyl alcohol (99.5%), purchased from Sigma-Aldrich (St. Louis, MO, US) and Samchun (Seoul, Korea), respectively, were employed for the crosslinking of the GO laminates. All of the organic dyes (rose bengal (RB, 1017 g mol^−1^), methyl blue (MB, 800 g mol^−1^), acid fuchsin (AF, 585.5 g mol^−1^), rhodamine B (RhB, 479 g mol^−1^), and methyl orange (MO, 327 g mol^−1^)) and ionic salts (sodium sulfate anhydrous (Na_2_SO_4_, >99.0%), sodium chloride (NaCl, >99.5%), magnesium sulfate anhydrous (MgSO_4_, >99.0%), and magnesium chloride hexahydrate (MgCl_2_·6H_2_O, >98.0%)) were purchased from Daejung Chemical (Si-heung, Korea). A nylon membrane filter was purchased from SciLab Korea Co., Ltd (Seoul, Korea) to synthesize GO. Other nylon filter (diameter: 47 mm, Millipore filters with a pore size of 0.2 μm; Saint-Quentin, France) was used as the support layers of the GO membranes.

### 2.2. Synthesis of GO

GO nanoflakes were synthesized by the modified Hummers’ method [31,32]. K_2_S_2_O_8_ (3 g) and P_2_O_5_ (3 g) were added to 30 mL of H_2_SO_4_ with mild stirring. After that, 2 g of graphite was slowly added to the solution, followed by stirring under a temperature-controlled condition at 80 °C for 12 h. Next, 500 mL of DI water was slowly added to the solution, followed by cooling to room temperature. The preoxidized graphite powder was obtained by drying at 45 °C for 24 h after passing the solution through a 47 mm membrane filter (nylon, pore size of 0.2 μm, SciLab Korea Co., Ltd., Seoul, Korea). The powder obtained from the previous step was added to 150 mL of H_2_SO_4_ with stirring. Thereafter, KMnO_4_ (12.5 g) was completely dissolved in the solution in an ice bath and subsequently heated at 35 °C for 12 h. DI water (2.5 L) was poured slowly into the solution as the temperature increased by more than 90 °C. After 30 min, 25 mL of H_2_O_2_ was added to the solution dropwise, and the dark brown color of the solution changed to yellow. The mixture was washed with a 10 wt% HCl solution and subsequently with DI water, followed by centrifugation at 3500 rpm and dialysis for 7 days to remove impurities. Finally, the GO suspension was obtained with evenly distributed functional groups.

### 2.3. Preparation of GO/DCX Membranes

A GO suspension with a concentration of 0.01 mg mL^−1^ was prepared and sonicated in a cool bath for 30 min to facilitate the homogeneous dispersion of GO in water. The GO solution (100 mL) was deposited on a nylon support membrane by a vacuum-assisted filtration method. The prepared GO membrane was dried at room temperature for 24 h [21]. Thereafter, a GO layer was crosslinked with DCX by immersing the as-prepared GO membranes in a DCX solution (2 wt%), prepared by dissolving DCX in methanol. To control the crosslinking degree, this crosslinking step was conducted at two different temperatures: room temperature and a high temperature (60 °C). The GO membranes crosslinked at room temperature and the high temperature are referred to as GO/DCX RT and GO/DCX HT, respectively.

### 2.4. Membrane Performance Test

The performance of the GO membranes was evaluated using a dead-end filtration system (Sterlitech HP4750, Auburn, WA, US) with an effective membrane area of 2.24 cm^2^ at a pressure of 1–5 bar. The pure water permeability (PWP) was measured when the PWP of the tested membrane reached a steady state after compaction, which took about 3 to 6 h. The PWPs were calculated using the equation:

(1) $$J=\frac{V}{\mathrm{At}\Delta p\;},$$

where J is the PWP (L m^−2^ h^−1^ bar^−1^ or LMH bar^−1^), V is the permeate volume (L), A is the effective area of the membrane (m^2^), t is the filtration time (h), and Δp is the applied pressure of the batch cell (bar).

The solute rejection efficiencies of the GO membranes were assessed with five organic solutions with concentrations of 10 mg L^−1^ (RB, MB, AF, RhB, and MO) and four different ionic solutions with a concentrations of 500 mg L^−1^ (Na_2_SO_4_, NaCl, MgSO_4_, and MgCl_2_). After the membrane performance reached the steady state, the samples for rejection analyses were collected after the additional operation for 6 h. The rejection efficiencies were computed using the following equation:

(2) $$R=\left(1-\frac{C_{p}}{C_{f}}\right)\times 100\;\%,$$

where R is the rejection of the solutes (%), C_p_ is the permeate concentration, and C_f_ is the feed concentration. The concentrations of the organic dyes and ionic solutions were determined using an ultraviolet–visible (UV–Vis) spectrophotometer (OPTIZEM Alpha, K LAB, Daejeon, Korea) and a conductivity meter (ST300C, OHAUS, Parsippany, NJ, US), respectively.

### 2.5. Membrane Characterization

The variations in the interlayer spacing, i.e., the nanochannel size, of the GO membranes were investigated by X-ray diffraction (XRD; Smart lab, RIGAKU, Austin, TX, US) with Cu–Kα_1_ radiation (wavelength: 0.154 nm). The morphologies of the GO membranes were characterized by scanning electron microscopy (SEM; SU8230, Hitachi, Tokyo, Japan). The chemical structure and composition of the membranes were examined by SEM–energy-dispersive X-ray spectroscopy (SEM–EDS; SU8230, Hitachi, Tokyo, Japan), Fourier-transform infrared spectroscopy (FT-IR; Nicolet iS50, Thermo Fisher Scientific, Waltham, MA, US), and X-ray photoelectron spectroscopy (XPS; Axis-Supra, Kratos, Manchester, UK). The membrane surface charge was determined using a zeta-potential analyzer (ELS-Z2, Otsuka, Osaka, Japan) in which a 0.01 M NaCl solution was employed as the electrolyte solution, and the surface hydrophilicity was measured using a contact-angle analyzer (Phoenix 300 Plus, SEO Co., Ltd., Suwon, South Korea) by dropping 5 µL of DI water on the membrane surface.

## 3. Results and Discussion

### 3.1. Characterization of the GO and GO/DCX Membranes

**Figure 1:**
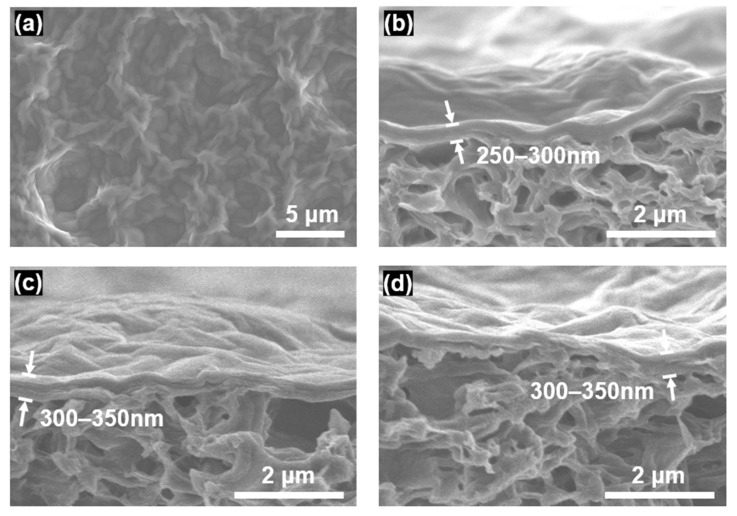
SEM images of (**a**) the surface of the pristine GO membrane and cross-sections of the (**b**) pristine GO, (**c**) GO/DCX RT, and (**d**) GO/DCX HT membranes.

Figure 1 shows microscopic images of the GO membranes. As shown in Figure 1a, the pristine GO membrane exhibited a corrugated surface, which is known to be typical for GO films formed on rough support layers [33]. The thickness of the pristine GO membrane was measured to be 250–300 nm by cross-sectional microscopic observation (Figure 1b). After the GO membranes were crosslinked with DCX, their thicknesses slightly increased to approximately 300–350 nm (Figure 1c for GO/DCX RT and Figure 1d for GO/DCX HT). This increased thickness implied that DCX was intercalated in between the GO nanosheets that constitute the GO membranes.

**Figure 2:**
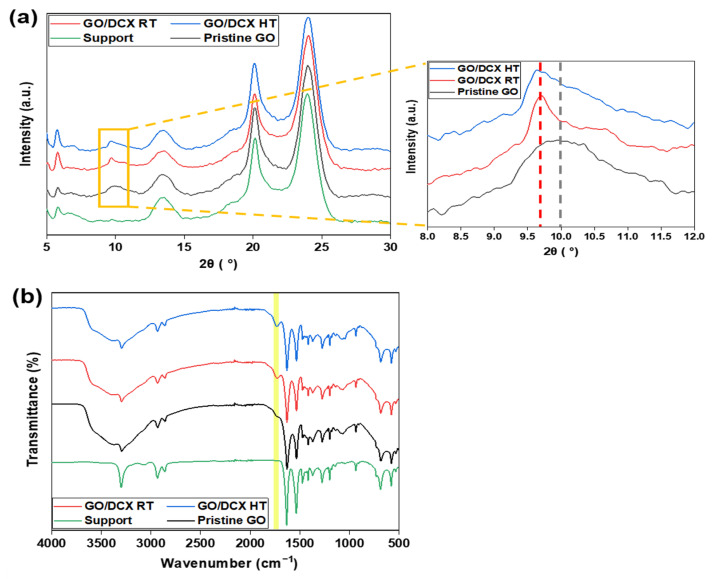
(**a**) XRD patterns of the prepared membranes and the magnified range of 8–12° to compare the differences in the interlayer distances of the GO membranes. (**b**) FT-IR spectra of the prepared membranes.

An XRD analysis was conducted to confirm the changes in the interlayer distance (i.e., the nanochannel size) of the GO nanosheets before and after the crosslinking reaction with DCX. As shown in Figure 2a, during the XRD analysis of the pristine GO membrane, a peak appeared at 9.94°, which was not observed during the analysis of the nylon support. This indicated that a laminar GO layer was successfully formed on a nylon support filter [34]. Conversely, for the cases of the two crosslinked GO membranes, the peaks shifted slightly to 9.7° and 9.64°, respectively. Such XRD peak shifts suggest that the interlayer spacing of the GO laminates increased from 0.889 nm to 0.911 nm and 0.916 nm in GO/DCX RT and GO/DCX HT, respectively.

**Figure 3:**
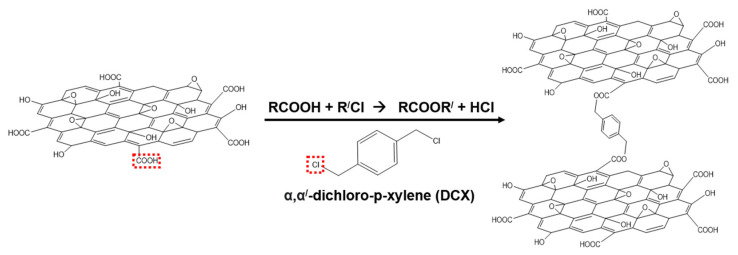
Mechanism of the crosslinking reaction between the graphene oxide (GO) nanosheets and α,α/-dichloro-p-xylene (DCX).

To further examine the effect of the DCX crosslinking on the GO membranes, the chemical structure of each membrane was analyzed by FT-IR (Figure 2b). Owing to the thin nature of the laminar GO layers, the absorption bands derived from the nylon support exhibited high intensities, even in the FT-IR spectra of the GO membranes. Consequently, there were no significant changes in the bands observed for some chemical bonds existing in both GO and nylon, such as C–O–C (1230 cm^−1^), C–OH (1417 cm^−1^), and C=C (1631 cm^−1^), after depositing the laminar GO layers. However, new bands for O–H (3303 cm^−1^), C=O (1736 cm^−1^), and C–O (1062 cm^−1^) appeared during the FT-IR analysis of the pristine GO membrane, confirming the presence of oxygen functional groups in the stacked GO layers. Meanwhile, in the spectra of the GO/DCX RT and GO/DCX HT membranes, the band for the C=O stretching vibration shifted from 1736 cm^−1^ to 1731 cm^−1^. The shift of the C=O stretching band probably occurred because some carboxyl groups (appearing in the range of 1710–1780 cm^−1^) in GO were substituted to ester groups (appearing in the range of 1730–1750 cm^−1^) via the crosslinking with DCX [35,36]. A detailed description of the reaction mechanism between the GO functional groups and DCX is presented in Figure 3. First, the carboxylic groups (–COOH) of the GO nanosheets reacted with the chloromethyl group (–CH_3_Cl) of DCX. Thereafter, the crosslinked DCXs with the paraxylene structure were positioned as support-column fixtures. Consequently, the DCXs were fixed between the stacked GO nanosheets.

The elemental composition and the binding conditions of each membrane were explored via EDS and XPS analyses. Based on the EDS analysis, it was found that the GO/DCX RT membrane had an increased atomic ratio of C and O (C/O ratio), attributed to the carbon atoms included in the DCX crosslink in the GO layer (Table 1). Moreover, the C/O ratio further increased in the GO/DCX HT membrane. This is presumably due to the thermally reduced GO layer and the increased DCX crosslinking degree. GO layers can be thermally reduced at a high temperature, resulting in the recovery of some sp^2^ graphitic structures and the removal of the oxygen functional groups in the GO layer. The surface color of the GO/DCX HT membrane changed to dark brown, confirming the reduction of its laminar GO layer (Figure S1). Additionally, at a high temperature, heat promoted the crosslinking reactions between DCX and GO, and consequently a relatively large quantity of DCXs reacted with the oxygen functional groups of the laminar GO layer.

In addition to the increase in the C/O ratio, the Cl atomic percentage was also affected by the DCX crosslinking. After the DCX crosslinking, a Cl atomic component with a meager percentage of 0.2% was detected during the EDX analysis of the GO/DCX RT membrane. This was due to the Cl atoms included in the unreacted halide groups of DCXs. This marginally detected Cl atomic component significantly increased when the GO layer was crosslinked at a high temperature, indicating that GO/DCX HT possesses many unreacted Cl atomic sites. Similar to the case of the C/O ratio increase, this could also be related to the reduction in the GO layer and the increase in the DCX crosslinking density. To begin with, the thermally reduced GO layer provided insufficient sites for the DCX crosslinking because of the decrease in the quantity of oxygen functional groups at high temperatures. Additionally, the heat-accelerated crosslinking reaction allowed a relatively large amount of DCX to react with the oxygen functional groups of the GO layer, which enhanced the crosslinking degree of the membranes while increasing the number of dangling DCX molecules via the reaction only at one end. This was corroborated by comparing the XPS results of the GO membranes.

**Figure 4:**
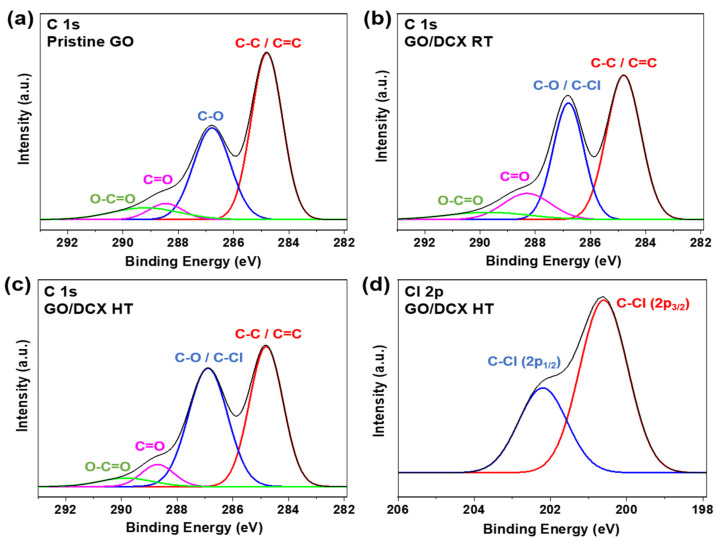
Deconvoluted C 1s XPS spectra of the (**a**) pristine GO, (**b**) GO/DCX RT, and (**c**) GO/DCX HT membranes and the (**d**) deconvoluted Cl 2p XPS spectra of the GO/DCX HT membrane.

Figure 4a–c show the deconvoluted C 1s XPS peaks of the GO membranes [37]. In the C 1s spectra of all the GO membranes, the deconvoluted peaks for the C–O bond were observed at 286.8 eV. From the C 1s spectra of the pristine GO membrane, the ratio of the C–O bond reached 34%. A similar ratio for the C–O bond (34.5%) was also observed in the C 1s XPS spectra of the GO/DCX RT membrane. However, a considerably higher ratio for the C–O bond (44.1%) was recorded in the C 1s spectra of the GO/DCX HT membrane. This could be explained by the overlapping of the C–O peak with the C–Cl peak at 286.8 eV. The presence of C–Cl bonds was further verified by the Cl 2p spectra (Figure 4d). The binding energy of organic chlorine was higher than 200 eV (Cl 2p_3/2_) [38], and thus, C–Cl bonds were present in the GO/DCX HT membrane, as indicated by the Cl 2p_3/2_ peak (200.6 eV). Due to the spin–orbit interaction, the binding energy of C–Cl 2p_1/2_ (202.2 eV) was 1.6 eV larger than that of C–Cl 2p_3/2_ (200.6 eV), and the area ratio of the two C–Cl 2p spectra was 1:2 [38,39]. This demonstrates that the GO/DCX HT membrane possesses many unreacted C–Cl bonds included in the DCX crosslinking networks.

**Figure 5:**
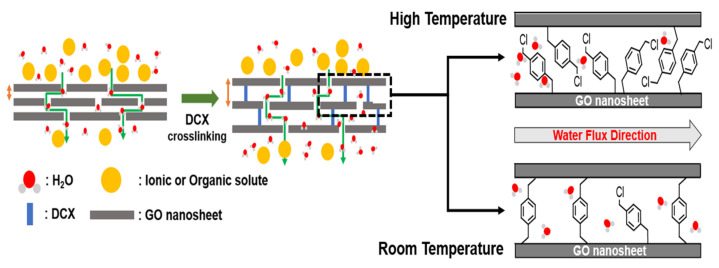
Schematic of the molecular transport in the GO laminate membranes with and without DCX crosslinking. The hypothetical nanochannel structures of the GO membranes crosslinked with DCX under room-temperature and high-temperature conditions are also shown on the right.

Based on the overall analysis of the characterization results, we can infer the effect of the DCX crosslinking on the structure of the GO membranes, as illustrated in Figure 5. First, the DCX crosslinking widened the interlayer spacing of the GO laminates. It is expected that the widened interlayer spacing facilitates the transport of water molecules across the GO nanochannels. Second, the temperature of the DCX crosslinking reactions may affect the crosslinking degree. At a high temperature, high-density crosslinks, along with many dangling DCXs possessing unreacted chloromethyl groups, are created in the GO nanochannels.

**Figure 6:**
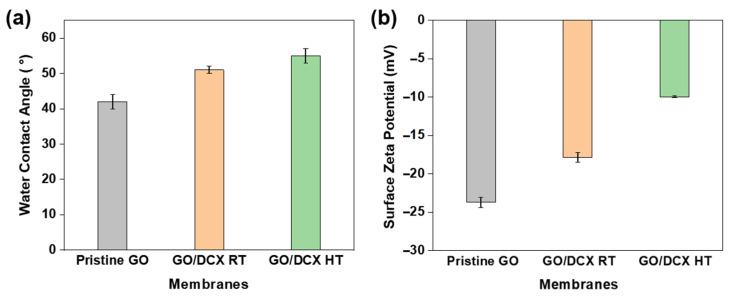
The (**a**) water contact angle and the (**b**) surface zeta potential of the prepared membranes.

Besides influencing the structure of the GO nanochannels, DCX crosslinking can also affect the critical surface properties of GO membranes. Owing to the intrinsic properties of GO nanosheets, GO-only membranes generally have a highly hydrophilic and negatively charged surface [40,41]. The water contact angle of the GO membranes increased from 42 ± 2° to 51 ± 1° when crosslinked with DCX at room temperature (Figure 6a). This is attributed to the nonpolar property of DCX. Furthermore, the GO/DCX HT membrane exhibited a relatively large water contact angle of 55 ± 2°, indicating its low hydrophilicity. The elimination of the oxygen functional groups or an increase in the DCX crosslinking degree due to the high temperature can lead to an increase in the hydrophobicity of the GO/DCX HT membrane surface [42,43]. Figure 6b presents the surface zeta potential of the GO membranes. The pristine GO membrane exhibited the most negatively charged surface, with a zeta potential of −23.7 ± 0.7 mV, followed by the GO/DCX RT membrane (−17.9 ± 0.7 mV) and the GO/DCX HT membrane (−10.0 ± 0.1 mV). The decreased surface charge of the GO/DCX membranes is also due to the DCX crosslinking and the GO layer reduction (Figure 6b) [41].

### 3.2. Molecular Separation Performance of the Membranes

**Figure 7:**
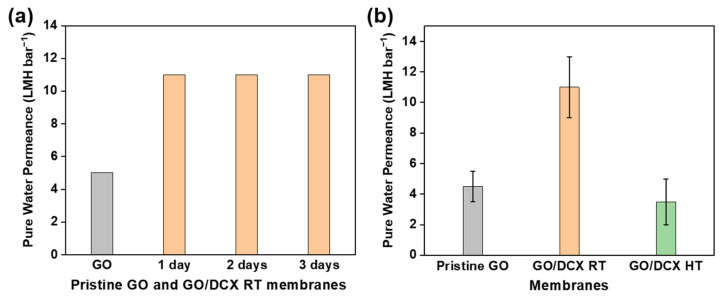
(**a**) Variations in the PWP of the GO membranes with DCX crosslinking time. (**b**) Comparison of the PWPs between the GO, GO/DCX RT, and GO/DCX HT membranes to determine the effect of temperature.

The effects of the DCX crosslinking on the performance of the GO membranes were examined by measuring the PWP of the prepared GO membranes using a lab-scale dead-end filtration system. As shown in Figure S2, the PWPs of the prepared GO membranes were almost proportional to the pressure in a specific range (1–5 bar). Figure 7a displays the effect of the DCX crosslinking time. The PWPs of all the DCX-crosslinked GO membranes were higher than those of the GO membrane without DCX crosslinking. This finding implies that the DCX crosslinked in GO nanochannels acts as an effective pillar that can create sufficient and aligned spaces to facilitate the flow of water molecules under pressurized conditions. This doubling of the PWP (11 ± 2 LMH bar^−1^) was pronounced at the crosslinking time of 1 d, and significant changes in the PWP were not observed at long crosslinking times, e.g., 2 d and 3 d. This result indicates that a 1 d duration can provide sufficient time for the crosslinking reaction between DCX and the oxygen functional groups of the GO nanosheets. Contrarily, when the DCX crosslinking occurred at a high temperature, the PWP of the GO/DCX membrane drastically decreased to 3.5 ± 1.5 LMH bar^−1^ (Figure 7b). As validated in a previous section, a high-temperature condition can lead to excessive high-density crosslinks, including dangling DCX molecules in the GO nanochannels, which may impede the flow of water.

**Figure 8:**
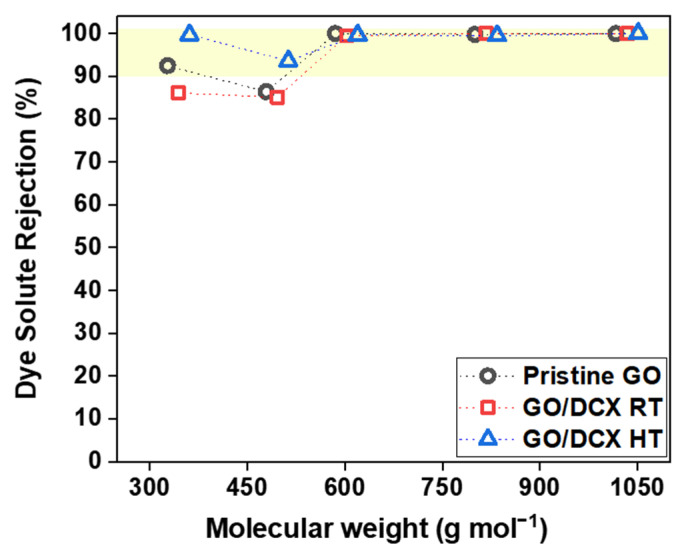
Molecular rejection efficiencies of the prepared GO membranes. All of the rejection tests were conducted with 10 mg L^−1^ concentrations of different types of organic dye molecules with molecular weights ranging from 327 g mol^−1^ to 1017 g mol^−1^ at an applied pressure of 1 bar for 12 h. The molecular weights of the chosen organic dye molecules are as follows: RB, 1017 g mol^−1^; MB, 800 g mol^−1^; AF, 585.5 g mol^−1^; RhB, 479 g mol^−1^; MO, 327 g mol^−1^.

Next, the solute-rejection performances of the prepared GO membranes were assessed. Figure 8 and Table S1 show the organic dye rejection efficiencies of the GO membranes for various organic dyes with different molecular weights. All the prepared GO membranes exhibited excellent rejection efficiencies of more than 99.5% for the targeted organic dye molecules with molecular weights of over 585.5 g mol^−1^. However, for the molecular weight of 479 g mol^−1^ (RhB), not only did the rejection efficiencies of these GO membranes decrease, but they also started to diverge. The GO/DCX RT membrane exhibited the most decreased RhB rejection efficiency of 85.1%, followed by 86.4% for the pristine GO membrane and 93.6% for the GO/DCX HT membrane. RhB is a charge-neutral molecule [44], and thus, it is believed that the size-based exclusion could work as a dominant mechanism for RhB rejection by these three GO membranes. Therefore, as confirmed in the XRD analyses, the nanochannels enlarged by the DCX crosslinking can result in the lowest RhB rejection efficiencies of the GO/DCX RT membrane. Conversely, the GO/DCX HT membrane exhibited the highest RhB rejection rate, although it also showed nanochannels widened by DCX crosslinking. This is likely due to the high DCX crosslink degree of the GO/DCX HT membrane. In contrast to the case with the water permeability, the high crosslink degree in the GO nanochannel can have a positive impact on solute rejection. This trend is also analogous to the rejection efficiencies for MO, which had the lowest molecular weight of 327 g mol^−1^. The highest rejection efficiency for MO was attained with the GO/DCX HT membrane, while the lowest was exhibited by the GO/DCX RT membrane. However, interestingly, despite MO having the lowest molecular weight (327 g mol^−1^) among the selected organic dyes, all of the GO membranes achieved higher rejection efficiencies for MO than they did for RhB. This is due to the Donnan exclusion effect. In addition to the size-exclusion effect, the electrostatically repulsive force generated between the same negatively charged MO molecules and the surface of the GO membranes caused more MO molecules to be rejected by the GO membranes.

Based on these results for organic-dye separation performance, it is clear that the GO/DCX RT membrane with moderately DCX-crosslinked nanochannels improves the water permeance without a significant loss of solute rejection. Conversely, the GO/DCX HT membrane with excessive DCX-crosslinked nanochannels containing many chloride-terminated tangling organic molecules is beneficial for highly selective separation.

### 3.3. Ionic Separation Performance of the Membranes

**Figure 9:**
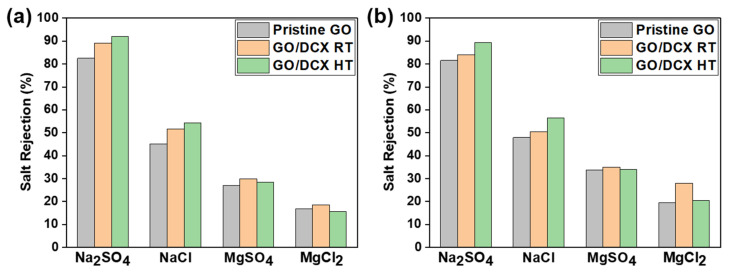
Salt rejection rates (Na_2_SO_4_, MgSO_4_, NaCl, and MgCl_2_) of the GO membranes at (**a**) 3 bar and (**b**) 5 bar.

To further evaluate the NF performance of the prepared membranes, salt-rejection tests were conducted with four common ionic solutions, Na_2_SO_4_, NaCl, MgSO_4_, and MgCl_2_, at different applied pressures of 3–5 bar. Figure 9a,b and Table S2 show the salt rejection efficiencies of the prepared GO membranes. The rejection efficiencies of the pristine GO membrane were compared according to salt type, and the observed order was Na_2_SO_4_ > NaCl > MgSO_4_ > MgCl_2_. Although the hydrated ionic size of Mg^2+^ (0.856 nm) is larger than that of Na^+^ (0.716 nm) [45], the Mg^2+^-containing salt solutions exhibited relatively low rejection efficiencies. This indicates that the mechanism of salt rejection by the pristine GO membrane is not dominated by size exclusion due to the small size of the ions, in contrast to the case with the organic-dye rejection. Rather, the Donnan exclusion effect governs salt rejection. This is evinced by the positive correlation between our experimental result and the theoretically predicted trend of salt rejection (Na_2_SO_4_ (2) > NaCl (1) ≈ MgSO_4_ (1) > MgCl_2_ (0.5)) based on the valence ratio of the cation and anion (Z^−^/Z^+^) [46,47]. During the filtration of the salt solutions, the negatively charged surface of the GO membrane (as shown in Figure 6b) repulsed the identically charged ions (i.e., anions). Meanwhile, counterions (i.e., cations) were also stoichiometrically excluded to retain the electrical neutrality of the solutions [48]. However, the MgSO_4_ rejection efficiencies are somewhat different from the theoretical prediction, which implies that the ionic sieving by the GO membranes is also affected by another factor besides the Donnan exclusion effect. Notably, such an exceptional trend of MgSO_4_ has been observed in some studies on laminar GO membranes [49,50,51]. Although further investigation is required to determine the correct reason for this exception, we presume that it might be related to the high charge density and strong interaction with the GO of the Mg^2+^ ion. Compared to Na^+^ ions, Mg^2+^ ions can create stronger bonds with the graphitic π-electrons of GO and even preferentially form complexes with the oxygen functional groups of GO (i.e., hydroxyl, epoxide, and carboxylate edge groups). This result is due to their high ionic charge and electronegativity [27,41] and reveals that the Mg^2+^ ions have a good ability to neutralize the negative surface charge of GO membranes [52]. Additionally, Mg^2+^ ions can reside for long periods in the GO nanochannels because of the strong interaction with GO and significantly enlarge the GO nanochannels because of their large hydrated radii [53]. The neutralized surface charge and the nanochannels of the GO membrane expanded by Mg^2+^ ions presumably accelerate the penetration of SO_4_^2−^ ions across the laminar GO layer, while the GO membrane filters the MgSO_4_ solution. Furthermore, despite the enlarged GO nanochannels, Mg^2+^-intercalated GO nanochannels exhibited weak water permeation. Mg^2+^ ions can attract and hold more water molecules inside the nanochannels than Na^+^ ions because of their higher charge density [27]. The high charge density promotes the preferential binding of water molecules with the intercalated cations over other water molecules. The effect of both the facilitated ion permeation and the suppressed water permeation by the Mg^2+^ ions might explain the lower MgSO_4_ rejection efficiency compared to NaCl.

For the GO/DCX RT and GO/DCX HT membranes, the same salt rejection trends were observed as with our GO membranes but at high rejection efficiencies. This demonstrates that the DCX crosslinking seldom affects the salt-rejection mechanism, although it could contribute to enhancing the salt rejection efficiencies by tightly locking the stacked GO layers. The laminar GO layer can significantly widen the nanochannels used by the hydrated cations intercalated into the laminar GO layer in salt solutions [27,53]. This can deteriorate the salt rejection efficiency. However, DCX crosslinking can prevent hydrated-cation-caused nanochannel swelling by holding adjacent GO nanosheets together. The increased NaSO_4_ and NaCl rejection efficiencies of the GO/DCX HT membrane can also be explained by the less swollen GO nanochannels, which are attributed to its relatively high crosslinking degree. Nevertheless, with MgSO_4_ and MgCl_2_, the GO/DCX HT membrane exhibited a slight decrease in the rejection efficiencies compared to the GO/DCX RT membrane. This is likely due to the effect of combining the lowest surface charge of the GO/DCX HT membrane with the high charge-neutralizing ability of Mg^2+^ ions. The significantly offset negative charge of the GO/DCX HT membrane by Mg^2+^ adsorption might increase the ion permeation by weakening the electrostatic interaction between the ion and the membrane.

**Figure 10:**
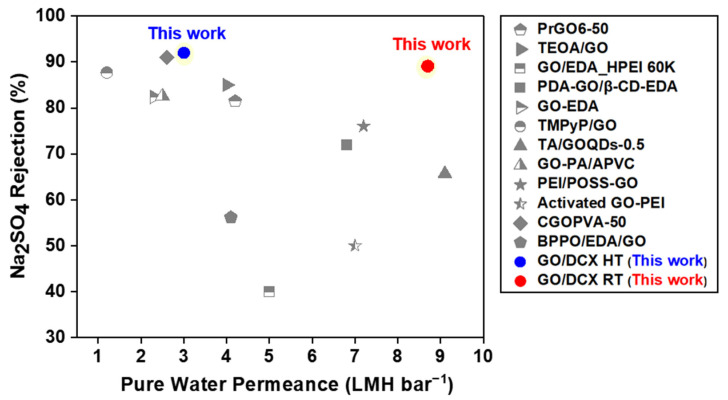
Performance comparison plot showing the Na2SO4 rejection efficiencies and the PWP of GO-based NF membranes, including our GO membranes [54,55,56,57,58,59,60,61,62,63,64,65].

Overall, we confirmed that DCX crosslinking can contribute to improving the salt-rejection efficiencies of GO membranes. To further evaluate the effect of DCX crosslinking, we also compared the NF performance of our DCX/GO membranes by benchmarking the Na_2_SO_4_ rejection and the PWP performances of previously reported crosslinked GO membranes (Figure 10 and Table S3) [54,55,56,57,58,59,60,61,62,63,64,65]. The results revealed that our GO/DCX membranes outperformed other crosslinked GO membranes. The GO/DCX HT membrane is positioned at the top for salt rejection. Moreover, the GO/DCX RT membrane is located at the upper-right position, which means the membrane beats the water permeation–rejection trade-off relation.

### 3.4. Long-Term Stability of the DCX-Crosslinked GO Membrane

**Figure 11:**
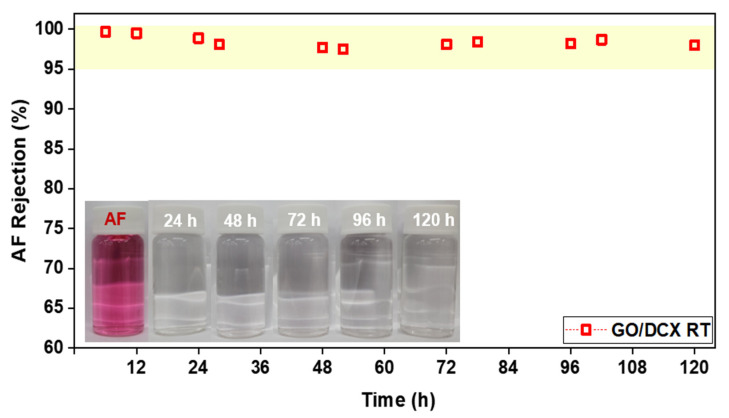
Monitoring of changes in the AF dye rejection efficiencies of the GO/DCX RT membrane during dead-end filtration for long-term stability evaluation (pressure: 1 bar; test duration: 120 h). The inset photograph shows the AF feed (10 mg L^−1^) and the permeates observed at 24 h intervals during the 120 h filtration test.

The effect of DCX crosslinking on the long-term stability of the GO membrane was assessed by continuously operating a dead-end filtration system equipped with the GO/DCX RT membrane with an AF solution, with vigorous stirring (250 rpm) for 120 h. Figure 11 shows the results of the AF rejection efficiencies monitored in 24 h intervals. The AF rejection efficiencies of the GO/DCX RT membrane were maintained at over 98% throughout the operation, indicating that no membrane damage or deterioration occurred. This result demonstrates that DCX crosslinking can effectively enhance the durability of GO-based NF membranes.

## 4. Conclusions

This study focused on modifying the interlayer spacing of GO laminates via DCX crosslinking. Through this interlayer-spacing adjustment, we successfully fabricated high-performance low-pressure GO-based NF membranes. The interlayer spacing of the modified GO membranes was enlarged by DCX crosslinking between the GO nanosheets to achieve a laminar GO layer, which was conducted at room temperature. The widened interlayer spacings contributed to the doubling of the PWP of the GO membranes by expediting the flow of water molecules. In addition, the dye rejection efficiency of the GO membranes was almost maintained without a tradeoff between the PWP and the solute rejection efficiency. When compared with previously reported crosslinked GO membranes, our DCX-modified GO membranes demonstrated superior performance. We developed a facile modification strategy, and the results confirm the application potential of GO for the development of high-performance low-pressure NF membranes.

## Figures and Tables

**Table 1:** Atomic percentage of each GO membrane obtained by EDS.

Membrane	Atomic Percentage (%) and Ratio			
	C	O	Cl	C/O
Pristine GO	79.4%	20.6%	0.0%	3.9
GO/DCX RT	86.6%	10.2%	0.2%	8.5
GO/DCX HT	91.7%	7.0%	1.3%	13.1

## Data Availability

Not applicable.

## Supplementary Materials

The following supporting information can be downloaded at https://www.mdpi.com/article/10.3390/membranes12100966/s1. Figure S1: Color change of the prepared membranes from dark blonde to dark brown; Figure S2: The pure water flux versus pressure (1−5 bar) of the prepared membranes; Table S1: Dye rejection efficiencies and PWP of the GO membranes at 1 bar; Table S2: Salt rejection efficiencies of the GO membranes under two different applied pressures; Table S3: Performance comparison of crosslinked GO-based NF membranes for water purification. References [54,55,56,57,58,59,60,61,62,63,64,65] are cited in the Supplementary Materials.
