# Supplementary material for: Graphene Oxide-Based Membranes Intercalated with an Aromatic Crosslinker for Low-Pressure Nanofiltration
Source: Membranes (Basel). 2022 Oct 2;12(10):966. doi: 10.3390/membranes12100966 (PMC9612350; doi:10.3390/membranes12100966)
Supplement: Supplementary file 1 [file membranes-12-00966-s001.zip › membranes-1941939-supplementary.pdf]

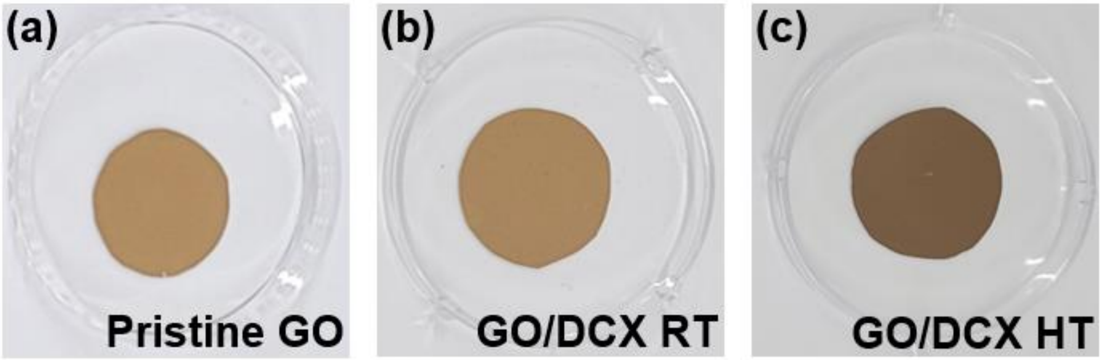

Figure S1. Color change of the prepared membranes from dark blonde ((**a**) Pristine GO and (**b**) GO/DCX RT) to dark brown ((**c**) GO/DCX HT).

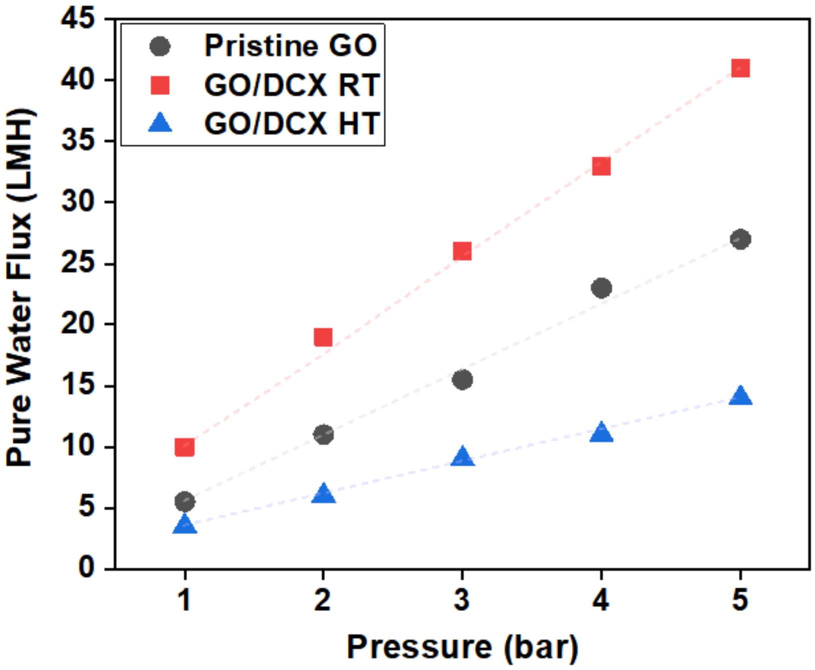

Figure S2. The pure water flux versus pressure (1−5 bar) of the prepared membranes.

Table S1 Dye rejection efficiencies and PWP of the GO membranes at 1 bar (feed solution concentration: 10 mg L^{-1}).

Membrane	Pure Water Permeance ($LMH\ bar^{-1}$)	Dye rejection rate (%)				
		RB	MB	AF	RhB	MO
Pristine GO	4.5 ± 1	100	99.8	100	86.4	92.5
GO/DCX RT	11 ± 2	100	100	99.5	85.1	86.1
GO/DCX HT	3.5 ± 1.5	100	99.5	99.6	93.6	99.7

Table S2 Salt rejection efficiencies of the GO membranes under two different applied pressures (feed solution concentration: 500 mg L^{-1}).

Membrane	Applied Pressure Δp (bar)	Salt rejection rate (%)			
		Na_2SO_4	NaCl	$MgSO_4$	$MgCl_2$
Pristine GO	3	82.5	45.2	26.9	16.8
	5	81.5	48.0	33.7	19.4
GO/DCX RT	3	89.1	51.6	29.9	18.6
	5	83.9	50.4	35.0	28.0
GO/DCX HT	3	92.0	54.4	28.3	15.6
	5	89.4	56.4	33.9	20.5

Table S3 Performance comparison of crosslinked GO-based NF membranes for water purification.

Membrane	Applied Pressure Δp (bar)	Pure Water Permeance (LMH bar^{-1})	Na_2SO_4		Ref.
			Concentration	Rejection (%)	
GO/DCX RT	3	8.7	500 ppm	89.1	This work
GO/DCX HT	3	3.0	500 ppm	92.0	This work
BPPO/EDA/GO	1	4.1	1000 ppm	56.2	[1]
CGOPVA-50	5	2.6 ± 1.1	20 mM	91.0	[2]
TEOA/GO	9	4	500 ppm	85.0	[3]
PDA-GO/β-CD-EDA	5	6.8	1000 ppm	71.9	[4]
Activated GO-PEI	1	7.0	2000 ppm	50.0	[5]
PEI/POSS-GO	4.5	7.2	1100 ppm	76.0	[6]
GO-PA/APVC	4	2.5	2000ppm	82.6	[7]
TA/GOQDs-0.5	2	9.1	1000 ppm	65.7	[8]
TMPyP/GO	8	1.2	2000 ppm	87.7	[9]
GO-EDA	2	2.3	500 ppm	82.4	[10]
GO/EDA_HPEI 60K	1	5	1000 ppm	40.0	[11]
PrGO6-50	8	4.2	500 ppm	81.5	[12]

Supplementary References

1. Meng, N.; Zhao, W.; Shamsaei, E.; Wang, G.; Zeng, X.; Lin, X.; Xu, T.; Wang, H.; Zhang, X. A Low-Pressure GO Nanofiltration Membrane Crosslinked via Ethylenediamine. *J. Membr. Sci.* **2018**, *548*, 363–371.
2. Park, M.J.; Nisola, G.M.; Seo, D.H.; Wang, C.; Phuntsho, S.; Choo, Y.; Chung, W.J.; Shon, H.K. Chemically Cross-Linked Graphene Oxide as a Selective Layer on Electrospun Polyvinyl Alcohol Nanofiber Membrane for Nanofiltration Application. *Nanomaterials* **2021**, *11*, 2867.
3. Nakagawa, K.; Araya, S.; Kunimatsu, M.; Yoshioka, T.; Shintani, T.; Kamio, E.; Matsuyama, H. Fabrication of Stacked Graphene Oxide Nanosheet Membranes Using Triethanolamine as a Crosslinker and Mild Reducing Agent for Water Treatment. *Membranes (Basel).* **2018**, *8*, 130.
4. Kong, F. xin; Liu, Q.; Dong, L. qian; Zhang, T.; Wei, Y. bin; Chen, J. fu; Wang, Y.; Guo, C. mei Rejection of Pharmaceuticals by Graphene Oxide Membranes: Role of Crosslinker and Rejection Mechanism. *J. Membr. Sci.* **2020**, *612*, 118338.
5. Parsamehr, P.S.; Zahed, M.; Tofighy, M.A.; Mohammadi, T.; Rezakazemi, M. Preparation of Novel Cross-Linked Graphene Oxide Membrane for Desalination Applications Using (EDC and NHS)-Activated Graphene Oxide and PEI. *Desalination* **2019**, *468*, 114079.
6. Bandehali, S.; Moghadassi, A.; Parvizian, F.; Zhang, Y.; Hosseini, S.M.; Shen, J. New Mixed Matrix PEI Nanofiltration Membrane Decorated by Glycidyl-POSS Functionalized Graphene Oxide Nanoplates with Enhanced Separation and Antifouling Behaviour: Heavy Metal Ions Removal. *Sep. Purif. Technol.* **2020**, *242*, 116745.

7. Qin, Y.; Liu, H.; Liu, Y.; Chen, M.; Chen, K.; Huang, Y.; Xiao, C. Design of a Novel Interfacial Enhanced GO-PA/APVC Nanofiltration Membrane with Stripe-like Structure. *J. Membr. Sci.* **2020**, *604*, 118064.

8. Zhang, C.; Wei, K.; Zhang, W.; Bai, Y.; Sun, Y.; Gu, J. Graphene Oxide Quantum Dots Incorporated into a Thin Film Nanocomposite Membrane with High Flux and Antifouling Properties for Low-Pressure Nanofiltration. *ACS Appl. Mater. Interfaces* **2017**, *9*, 11082–11094.

9. Xu, X.L.; Lin, F.W.; Du, Y.; Zhang, X.; Wu, J.; Xu, Z.K. Graphene Oxide Nanofiltration Membranes Stabilized by Cationic Porphyrin for High Salt Rejection. *ACS Appl. Mater. Interfaces* **2016**, *8*, 12588–12593.

10. Su, J.; Jia, M.; Wu, W.; Li, Z.; Li, W. Chemical Vapor Crosslinking of Graphene Oxide Membranes for Controlling Nanochannels. *Environ. Sci. Nano* **2020**, *7*, 2924–2929.

11. Zhang, Y.; Zhang, S.; Chung, T.S. Nanometric Graphene Oxide Framework Membranes with Enhanced Heavy Metal Removal via Nanofiltration. *Environ. Sci. Technol.* **2015**, *49*, 10235–10242.

12. Zhang, Z.; Li, N.; Sun, Y.; Yang, H.; Zhang, X.; Li, Y.; Wang, G.; Zhou, J.; Zou, L.; Hao, Z. Interfacial Force-Assisted In-Situ Fabrication of Graphene Oxide Membrane for Desalination. *ACS Appl. Mater. Interfaces* **2018**, *10*, 27205–27214.
